# Evaluation of host immune responses to Mycobacteriophage Fionnbharth by route of delivery

**DOI:** 10.1186/s12985-024-02552-2

**Published:** 2025-01-20

**Authors:** Thomas Smytheman, Tiffany Pecor, Dana E. Miller, Debora Ferede, Suhavi Kaur, Matthew H. Harband, Hazem F. M. Abdelaal, Carlos A. Guerrero-Bustamante, Krista G. Freeman, Whitney E. Harrington, Lisa M. Frenkel, Graham F. Hatfull, Rhea N. Coler, Sasha E. Larsen

**Affiliations:** 1 Center for Global Infectious Disease Research, Seattle Children’s Research Institute, Seattle, WA USA; 2 Department of Biological Sciences, University of Pittsburgh, Pittsburgh, PA USA; 3 Department of Pediatrics, University of Washington, Seattle, WA USA; 4 Department of Global Health, University of Washington, Seattle, WA USA

**Keywords:** Mycobacteriophage, Tuberculosis, Humoral immunity, Aerosol delivery

## Abstract

For much of the last decade, tuberculosis (TB) was the leading cause of mortality due to an infectious pathogen (*Mycobacterium tuberculosis*, M.tb). Approximately 1.3 million deaths in 2023 worldwide were attributed to TB disease. Focused intervention strategies to block transmission would significantly reduce the global health burden of TB. Mycobacteriophages (phages) are a sorely underutilized biologic therapy for the pathogen M.tb, and here we aimed to address outstanding questions about their utility for clinical applications. We aimed to determine the impact of repeated mucosal or intravenous (IV) delivery of representative anti-M.tb phage FionnbharthΔ45Δ47 (Fionnbharth) in a preclinical mouse model. In addition, we specifically sought to understand which route induced anti-phage antibodies, which may reduce the long-term impact of phage therapy. C57BL/6 mice were dosed weekly for 6 weeks by either route and serum and bronchoalveolar lavage fluid (BALf) were evaluated for anti-phage humoral responses by ELISA. We found that aerosol delivery disperses phage across all lung lobes where M.tb is also found after experimental infection by the same route. Repeated aerosol delivery was well tolerated and did not induce robust neutralizing humoral immunity. In contrast, Mice receiving IV phage developed increasing magnitude and neutralizing total IgG and IgA responses over time. To determine whether pre-treatment environmental exposure to Fionnbharth-like phages could induce antibody responses that are potentially neutralizing, ~ 500 human plasma samples from normal donors were evaluated by ELISA. We observed that 5% of samples had antibodies to Fionnbharth (with end point titers > 10^− 3^ dilution), although none were neutralizing. Furthermore, we found that highly-purified phage preparations did not activate mouse or human derived toll like receptor (TLR) 4 or TLR9 in HEKblue reporter assays. These data together support using Fionnbharth in anti-M.tb therapy phage cocktail strategies and that aerosol delivery should be prioritized for further efficacy testing.

**Supplementary Information:**

The online version contains supplementary material available at 10.1186/s12985-024-02552-2.

## Introduction

Before the COVID-19 pandemic, pulmonary tuberculosis disease (TB) caused by *Mycobacterium tuberculosis* (M.tb) was the leading infectious killer globally [1]. Approximately a quarter of the global population is estimated to be latently infected with TB, with more than 10 million new cases each year [1]. The Center for Disease Control (CDC) estimates that recent transmission accounts for roughly 13% of TB cases in the U.S., representing an important point of intervention to combat the TB epidemic [2, 3]. Vaccination against M.tb is an active area of research and indeed much of the global community receives *Mycobacterium bovis* bacille Calmette-Guérin (BCG) vaccination against TB in infancy. While BCG can afford protection against severe disseminated mycobacterial disease in children, it provides limited to no prophylactic effect for adults and does not prevent M.tb infection or transmission [4–8]. A novel vaccine that only prevents transmission of M.tb could reduce infection incidence by 68% by mathematical modeling [9], however licensure of a potential candidate is years in the future and more tools for disrupting and treating infections are needed now.

Bacteriophage (phage) therapy is a promising regimen that may limit the spread of M.tb irrespective of drug resistance with the potential for population level-impacts. Case reports of intravenous (IV) phage therapy for nontuberculous mycobacterial (NTM) infections, arguably the most invasive intervention strategy, are strongly positive and show good safety profiles in emergency human clinical use [10, 11]. In addition to phage therapy for tuberculosis, a phage-based pre- or post-exposure prophylactic could potentially be used to reduce M.tb transmission, especially in instances with known exposures. Previous work has demonstrated a reduction in M.tb colony forming units (CFU) in mice when mycobacteriophages are delivered via aerosol prior to M.tb infection [12]. However, much remains unknown about the feasibility and biodistribution of phages delivered via different administrative routes and the subsequent host humoral immunity that may be induced. Here we characterize and compare the delivery mode of a lytic phage derivative, FionnbharthΔ45Δ47, that is broadly-active against M.tb with therapeutic potential in a mouse model system [11, 13, 14]. We evaluated the immune response dynamics after weekly repeated dosing, as well as pharmacokinetics of clearance from the lung post-delivery. We hypothesize that phages delivered via aerosol will provide better lung coverage and induce less anti-phage humoral immunity compared to IV delivery [15].

## Materials and methods

### Fionnbharth phage preparation

The phage was purified from bacterial lysate by double density gradient centrifugation in Cesium Chloride (CsCl) [16]. Briefly, the phage was concentrated by centrifugation of 200–500 mL of crude lysates (1 × 10^11^ PFU/mL) at 100,000 x g for 1 h. The phage pellets are reconstituted in 10 mL phage buffer (10 mM Tris-HCl (pH 7.5), 10 mM MgSO_4_, and 68 mM NaCl) with 0.85 g/mL CsCl and centrifuged at 135,000 x g for 16 h. The phage band is extracted from the cesium gradient and again resuspended in 10 mL of phage buffer with 0.85 g/mL CsCl, the density gradient centrifugation step is repeated. The CsCl was subsequently removed from the purified phage by dialyzing in phage buffer in 15 KD molecular weight cutoff dialysis membrane (Spectrum Scientific). The dialyzed phage was then diluted in sterile 0.9% saline (Fisher Scientific).

### Mouse model of M. tuberculosis and phage delivery

Female C57BL/6 mice 4–6 weeks of age were purchased from Charles River Laboratory. The mice were housed at the Seattle Children’s Research Institute (SCRI) biosafety level 3 animal facility under pathogen-free conditions when infected with M.tb. and biosafety level 1 for repeated dosing with phage without M.tb infection. Animals were handled in accordance with the specific guidelines of the SCRI Institutional Animal Care and Use Committee. The institute operates under USDA Certificate #91-R-0061 and PHS Assurance #A4337-01. Mice were infected with a low-dose (~ 25 bacteria) aerosol (LDA) of M.tb H37Rv (BEI Resources, NR-13648) using a GlasCol^®^ Inhalation Exposure System. Twenty-four hours post-challenge, the lungs of 10 mice were isolated and individual lung lobes were homogenized using an Omni Tissue Homogenizer in CFU buffer (PBS + 0.05% Tween80) then plated on Middlebrook 7H11 agar (Fisher Scientific) to enumerate colony forming units (CFU). Lobes were identified as the following: Lobe 1 – Post-Caval Lobe; Lobe 2 – Right Superior Lobe; Lobe 3 – Right Middle Lobe; Lobe 4 – Right Inferior Lobe; Lobe 5 – Left Lung.

C57BL/6 mice were dosed with purified phage Fionnbharth via inhalation or IV injection. Aerosol dosing was performed using the InExpose nose-only inhalation system (SciReq) in which a 7.5 × 10^10^ Plaque Forming Units (PFU)/mL Fionnbharth solution was nebulized with an Aerogen mesh vibrating nebulizer and delivered to the chamber for 20 min. Intravenous doses were administered via tail vein injections of 200 µL of a 1 × 10^9^ PFU/mL Fionnbharth solution. Fionnbharth delivery to the lungs was evaluated after dosing methods using the plaque assay with a top lawn of *M. smegmatis* mc^2^155 as previously described [17, 18]. At 30 min, 1, 3, 6, 24 and 48 h after Fionnbharth dosing, mice were sacrificed, and individual lung lobes (as described above) were collected and homogenized separately in phage buffer. The homogenized samples were then serially diluted in phage buffer to be plated within the Top Agar. A total of 10 µL of each dilution was combined with 0.5 mL of *M. smegmatis* mc^2^155 (OD_600_ 1.0) and left for 20 min before being mixed with equal volumes of Middlebrook Top Agar (MBTA) and 7H9 (Fisher Scientific) + 2 mM CaCl_2_ to form the Top Agar overlay (0.35% agar) on a 0.2% glucose 7H10 agar plate. After 24 h of incubation at 37 °C, plaques were counted.

After 2, 4, or 6 doses of IV or aerosol Fionnbharth (1 × 10^9^ PFU/mL) mice were sacrificed for serum and bronchoalveolar lavage fluid (BALf) collection. Serum was obtained via retro orbital blood collection and BALf was collected by washing the airways with 2.0 mL of PBS two times and combining the resultant fluid returned. C-reactive protein (CRP) levels in mouse serum were evaluated over the weekly IV or aerosol dosing regimen. CRP quantities were determined using the Mouse CRP ELISA kit (abcam Cat # ab222511) according to manufacturer instructions. In a separate study, Fionnbharth phage was administered weekly via tail vein injections of 200 µL of a 1 × 10^9^ PFU/mL, 1 × 10^6^ PFU/mL, or 1 × 10^4^ PFU/mL Fionnbharth solution, diluted in sterile saline and serum and BALf samples were collected after 5 or 6 doses.

### HEK-blue reporter cells

Human and mouse derived TLR4 and TLR9 HEK-Blue reporter cells (InvivoGen) were expanded in complete DMEM (4.5 g/L glucose, 2 mM L-glutamine, 10% (v/v) fetal bovine serum, 100 U/mL penicillin, 100 µg/mL streptomycin, 100 µg/mL Normocin) and seeded at 1 × 10^6^ cells/mL in a flat bottom 96-well plate where 150 µL were added to each well and cultured overnight at 37 °C + 5% CO_2_. The following day each cell type (hTLR4, mTLR4, hTLR9, mTLR9) was either left untreated, treated with 100 ng/mL of LPS (Millipore Sigma), 25 µg/mL of CPG oligonucleotide 2216 (Miltenyi Biotec), or 10^9^ PFU/mL of Fionnbharth in triplicate with HEK-Blue Detection media (InvivoGen) for detection of secreted embryonic alkaline phosphatase (SEAP). The colorimetric readout was performed after 24 h of stimulation at an optical density (O.D) of 620–655 nm on a SpectraMax iD3 (Molecular Devices) plate reader. Raw O.D. values were normalized to the average of the untreated wells.

### Predictive immunogenicity – IEDB

The Fionnbharth amino acid sequence was obtained from GenBank (JN831653) and determined to contain 32 annotated genes and 93 hypothetical proteins. Seven tail structural proteins were prioritized and analyzed via the Immune Epitope Database & Tools (IEDB) platform. Major histocompatibility complex (MHC) Class I binding predictions were run on 1/24/2024 using the IEDB analysis resource NetMHCpan (ver 4.1). A 27-allele reference panel covering ~ 97% of the human population was used to determine a binding score for all possible peptides (peptide length 9) of the 7 identified tail structural proteins. Peptide and allele combinations with a binding score above the 90th percentile were then analyzed using the IEDB Class I Immunogenicity resource (http://tools.iedb.org/immunogenicity/reference/*).* The MHC Class II binding predictions were made on 2/7/2024 using the IEDB analysis resource NetMHCIIpan (ver. 4.1) tool. A 27-allele reference panel covering ~ 97% of the human population was used to determine a binding score for all possible peptides (peptide length 15) of the 7 identified tail structural proteins. B Cell Linear Epitope Predictions were run on 2/14/2024 using the Bepipred Linear Epitope Prediction (V. 2.0 for proteins less than 250 amino acids long, V 1.0 for proteins with more than 250 amino acids long). V 2.0 of the program has a default threshold of 0.5, and a threshold of 0.5 was selected for proteins analyzed using V 1.0 where the default is 0.350. Peptides identified as having predicted immunogenic epitopes were compared between T Cell and B Cell assessments to identify those that were identified in both analyses.

### Human plasma samples

Human plasma samples were collected as part of a 52 week-long prospective COVID-19 detection study from April 2020 – July 2021 from employees at Seattle Children’s Research Institute and their household members, under Seattle Children’s IRB (STUDY00002434) [19]. The average donor age was 35.6 years old and ranged from 1 to 80. The donor composition was 54% female and the total *n* = 498.

### Enzyme linked immunosorbent assay (ELISA)

An in-house 384-well high throughput ELISA was developed to detect anti-Fionnbharth Total IgG, IgA mouse and human humoral responses. Details described below.

#### Coating

Sterile High Binding 384 well plates (Corning, Corning, NY) were filled with 50 µL per well of ELISA coating buffer containing Fionnbharth phage with a final concentration of 2 × 10^9^ PFU/mL. Coating buffer was made in house using one standard packet of ELISA coating buffer powder (ebioscience) mixed with 1 L of distilled water and filtered with a 0.22 μm filter to generate 1 L of 0.01 M PBS, pH 7.4. Plates were incubated for at least 2 h at room temperature (RT), or up to 3 days at 4 °C.

#### Blocking

Wash Buffer A was made by diluting 1 L of 20× Wash Buffer A solution (Teknova) in 19 L of H_2_O from a Barnstead Nanopure Water system (Thermo Scientific). After incubation with Fionnbharth, plates were washed three times with 100 µL per well of 1× Wash Buffer A. Plates were washed using a BioTek EL406 plate washer (BioTek). For mouse sample plates, blocking buffer was made by adding 10.0 g dry powdered milk (Walmart) in 1000 mL of PBS with 0.05% Tween. For human sample plates, blocking buffer was made by adding 10.0 g bovine serum albumin (Sigma) in 1000 mL PBS with 0.05% Tween. Each well received 80 µL of blocking buffer for all plates. Plates were incubated for at least 2 h at RT, or at 4 °C overnight.

#### Sample addition and dilution

Mouse serum and BALF plates were then washed three times with 1× Wash Buffer A and subsequently 50 µL of diluent (mixture of 100 mL of blocking buffer in 900 mL of 1x Wash Buffer A) was added to every well of the washed plates. Next, mouse serum samples were diluted 1:2 in diluent in a 96-well master block before their addition to the 384-well ELISA plate and subsequently diluted 1:5 from left to right across the plate in a 12-point dilution, discarding 12.5 µL from the final dilution column. For mouse BALf samples, following the washing, 50 µL of diluent was added to each well except for the initial well of the 12-point dilution series. BALf samples were manually pipetted into the first column of each dilution series and subsequently diluted 1:5, discarding 12.5 µL from the final dilution column. For human plasma samples, plates were washed three times with 1× Wash Buffer A and subsequently 50 µL of diluent (mixture of 1.0 g bovine serum albumin, 500 mL 1× Wash Buffer A and 500 mL PBS) was added to every well of the washed plates. Next, plasma samples were diluted 1:10 in diluent (10 µL of plasma to 90 µL of diluent) in a 96-well master block before their addition to the 384-well ELISA plate. 12.5 µL of the master block sample dilution was pipetted into the first column of each sample and subsequently diluted 1:5 for an 8-point dilution starting at 1:100, discarding 12.5 µL from the final dilution column. Plates were incubated overnight at 4 °C.

#### Development

After incubation, plates were removed from the refrigerator and left to warm to RT. Once warmed, plates were washed five times with 1× Wash Buffer A. Horseradish peroxidase (HRP) conjugated Goat Anti-Mouse IgG antibodies (Southern Biotech Cat # H0021-QC93) were diluted (diluent mixture described above) by a factor of 4000, HRP conjugated Goat Anti-Mouse IgA antibodies (Southern Biotech Cat # C0919-P0510) were diluted by a factor of 2000, HRP conjugated human rec-Protein G antibodies (Invitrogen, 101223) were diluted by a factor of 4000 and HRP-conjugated anti-human IgA antibodies (Invitrogen, PA1-74395) were diluted by a factor of 2000. Plates were incubated for 1 h in the dark at RT, then washed five times with 1× Wash Buffer A followed by one wash with 1× PBS. Plates then received 50 µL of Tetramethylbenzidine (TMB) (SeraCare, Milford, MA) per well. After 1 min 40 s of TMB incubation for mouse IgG, 5 min for mouse and human IgA, and 2 min 40 s for human IgG, 25 µL of 1 N H_2_SO_4_ (Sigma) was added to each well to halt the TMB reaction. Plates were read at a wavelength of 450 nm with a reference filter set at 570 nm using a SpectraMax iD3 plate reader (Molecular Devices) and SoftMax Pro 7.1.2 analysis software.

#### End point titer (EPT) calculation

In mouse samples, the O.D. value was calculated using 450 nm minus 570 nm values and the average O.D. value for the negative control (serum from mouse dosed IV with unrelated mycobacteriophage) was used to set a minimum cutoff value for each plate. These plate cutoff values were then used to calculate each sample EPT using a least squares regression model from GraphPad Prism. Human sample O.D. values were calculated using 450 nm minus 570 nm values and the average O.D. value for the negative control (lowest responder from human plasma ELISA development pilot) was used to set a minimum cutoff value for each plate. These plate cutoff values were then used to calculate each sample EPT using a 4-parameter logistic model in XL-fit software (model 208) as a Microsoft Excel add-in.

### Neutralization assay

Mouse serum samples were diluted (1:10) in phage buffer and incubated with Fionnbharth phage (1 × 10^9^ PFU/mL) for 24 h at RT. Next, 10-fold serial dilutions were made and 10 µL of each dilution was mixed into top agar overlays of *M. smegmatis* as described above and then incubated at 37 °C for 24 h for the ‘full plate neutralization assay’. Plaques were then counted to quantify the amount of active phage remaining after incubation with serum where loss in phage plaques observed were considered due to neutralizing antibodies as previously described [15]. In a separate series, denoted as ‘stamp neutralization assays’, mouse serum or human plasma samples were diluted (1:10) in phage buffer supplemented with 16% ADC and then diluted in a 3-fold serial dilution in a 96-well plate. Fionnbharth phage was then diluted to 1 × 10^6^ PFU/mL in the same ADC supplemented phage buffer and added to each well for co-incubation at RT for 24 h. The next day, 2.5 µL of each co-incubation was spotted onto top agar overlays of *M. smegmatis* mc^2^155 in single well rectangle plates using a VIAFLO stamper (Integra) and then incubated at 37 °C for 24 h. The neutralizing titer is defined as the last serum dilution that yields no active phage plaques.

### Statistical analysis

Assessments made between groups over time (with 2 or more timepoints) were performed using 2-way ANOVA tests with Šidák’s multiple comparisons, including PFU pharmacokinetics and total IgG EPT over time. When analyzing Fionnbharth-specific IgA EPTs in Serum and BALf after 6 weekly aerosol or IV doses, statistical significance was assessed with an unpaired T-tests with Welch correction using the Holm-Šidák method. Statistical significance for Serum Neutralization Assay between untreated control samples and two phage treated cohorts at a single timepoint was determined using a one-way ANOVA and Dunnetts multiple comparisons test. Evaluations of CRP differences over time were evaluated by 2-way ANOVA tests with Šidák’s multiple comparisons between groups and Dunnetts comparisons within groups. Simple linear regressions were used to determine the relationship between anti-Fionnbharth total IgG and IgA responses in human plasma samples as well as the relationship between anti-Fionnbharth total IgG and donor age.

## Results

**Fig. 1:**
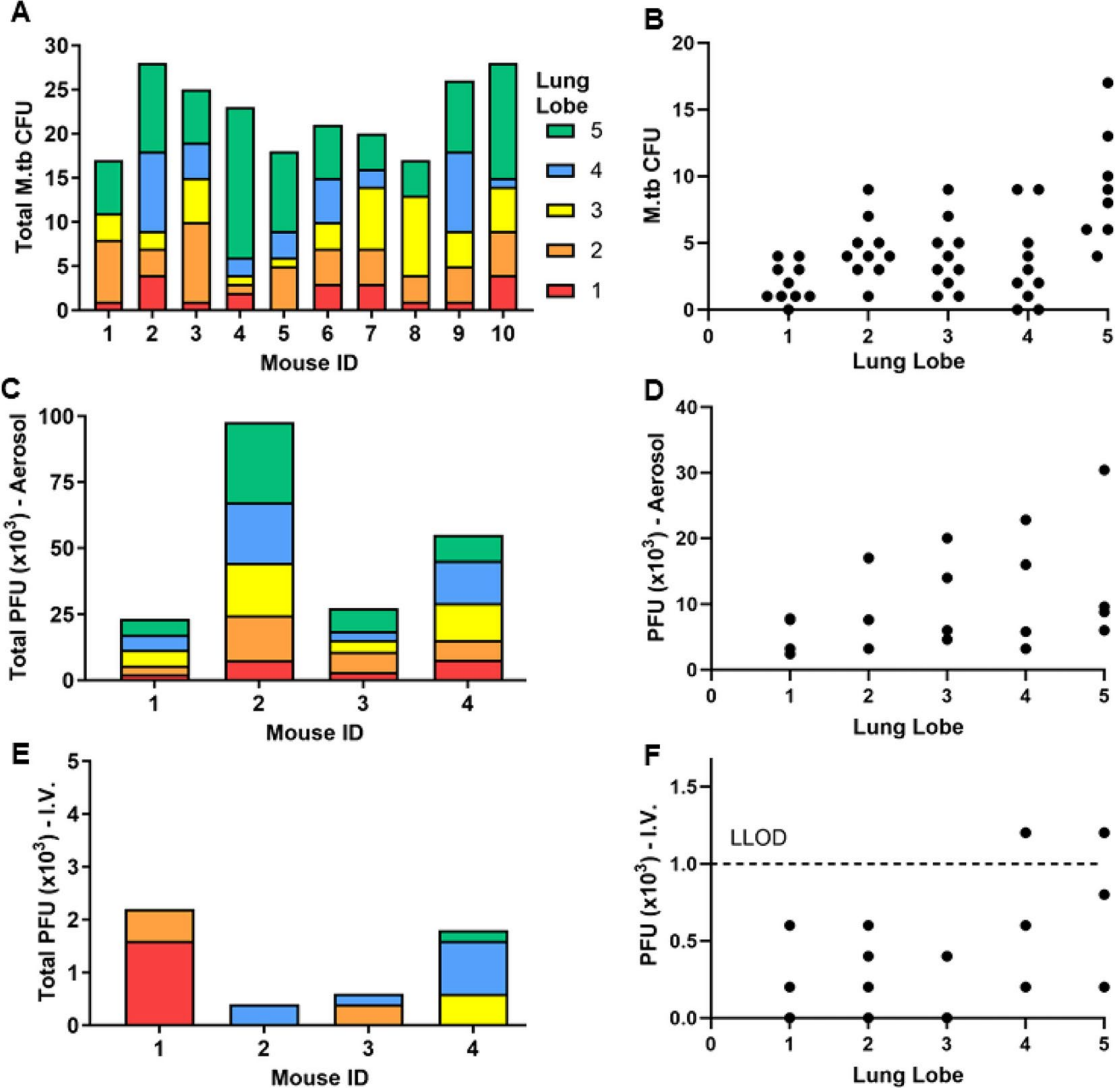
Characterization of phage deposition with different dosing routes. C57BL/6 mice were challenged with M.tb or separately dosed with aerosol or IV phage. M.tb and phage distribution across individual lung lobes where Lobe 1 – Post-Caval Lobe (red); Lobe 2 – Right Superior Lobe (orange); Lobe 3 – Right Middle Lobe (yellow); Lobe 4 –Right Inferior Lobe (blue); Lobe 5 – Left Lung (green) were performed using lung homogenates. Deposition of M.tb 24 h post-infection is shown as (**A**) total bacterial counts per mouse (*n* = 10) divided by individual lung lobes, and as (**B**) distribution of CFU per lung lobe across all mice. Deposition of Fionnbharth phage 30 min to 1 h after aerosol delivery is shown as (**C**) total PFU counts per mouse (*n* = 4) divided by individual lung lobes, and as (**D**) distribution of PFU per lung lobe across all mice. Deposition of Fionnbharth phage 30 min to 1 h after IV delivery is shown as (**E**) total PFU counts per mouse (*n* = 4) divided by individual lung lobes, and as (**F**) distribution of PFU per lung lobe across all mice. PFU were determined via *M. smegmatis* plaque assay using a dilution of lung homogenates

Mice challenged with low dose aerosols (LDA) of M.tb had bacilli well distributed across all lung lobes and in 9/10 mice all 5 lung lobes harbored M.tb at 24 h post-infection (Fig. 1A). Not surprisingly, the relative amount of M.tb was highest in the largest lung lobe (#5 entire left lobe) (Fig. 1B). In order for phage to be efficacious against M.tb, the two must colocalize in vivo, so we next evaluated the pulmonary distribution of Fionnbharth phage 30 min to one hour after aerosol (Fig. 1C, D) or IV (Fig. 1E, F) delivery. The distribution pattern of Fionnbharth phage delivered via aerosol was concordant with the distribution pattern of M.tb, where viable phage particles could be found in each lung lobe with a trend towards most being found in lobe 5 (Fig. 1C, D). Conversely, phage delivered IV was inconsistently distributed across lung lobes, with no more than 3 lobes harboring phage post-delivery per mouse (Fig. 1E, F). The amount of phage distributed was substantially higher when delivered via aerosol, with over a 10-fold difference in recovery from the lungs of treated mice (Fig. 1C, E).

**Fig. 2:**
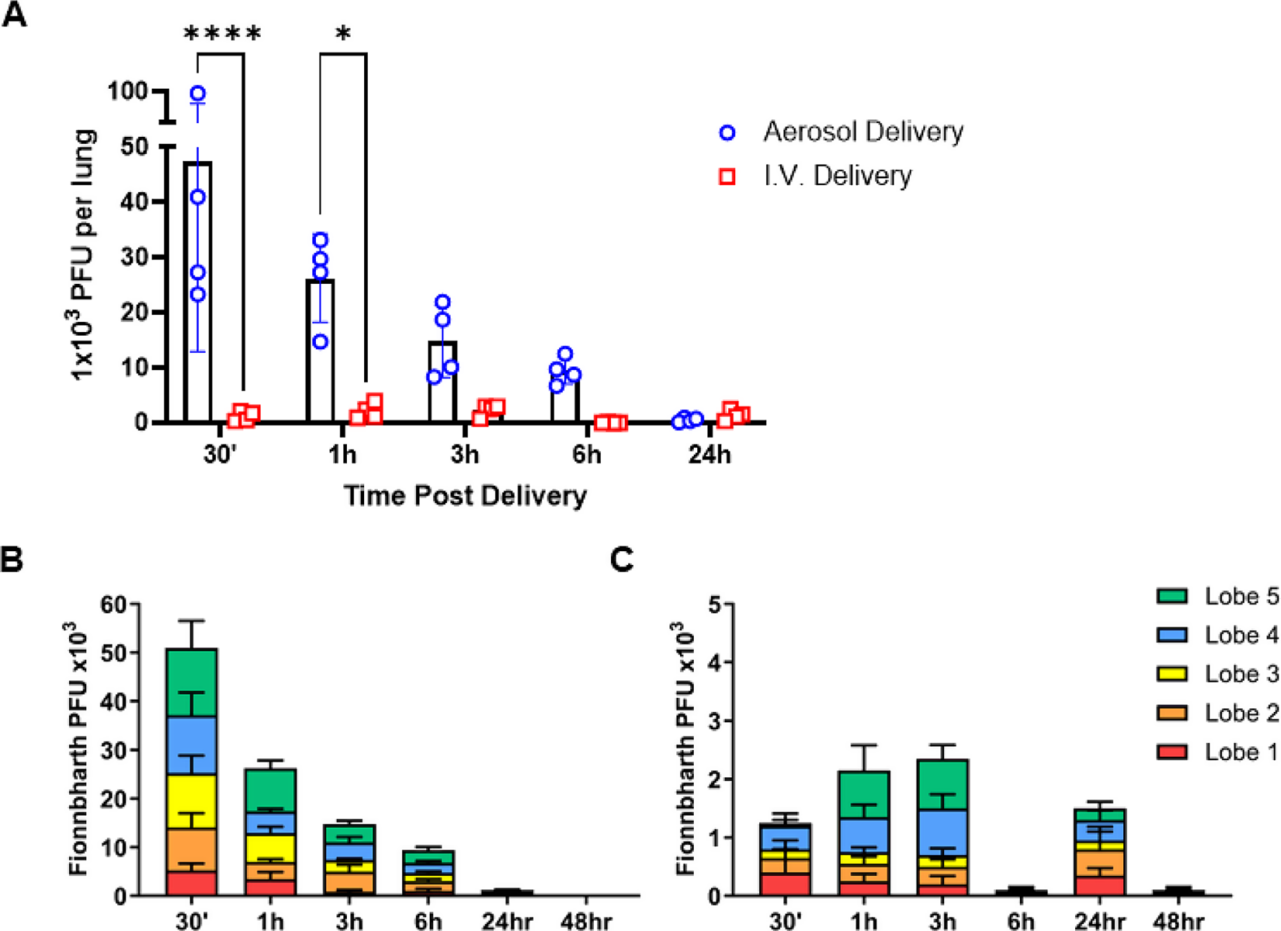
Pharmacokinetics of phage clearance after aerosol and IV dosing. C57BL/6 mice were given phage by aerosol or IV delivery and assessed for PFU at different times post-delivery. (**A**) Cumulative Fionnbharth PFU recovered per mouse (*n* = 4 per group, per timepoint) at 30 min, 1, 3, 6 and 24 h post aerosol (open blue circles) or IV (open red squares) delivery. Cohorts were compared via 2 way ANOVA and Šidáks correction, asterisks denote significant differences between delivery groups at the timepoint tested where **** *p* < 0.0001 and *p = 0.0273. Fionnbharth PFU per lung lobe over time after (**B**) aerosol, or (**C**) IV delivery where Lobe 1 – Post-Caval Lobe (red); Lobe 2 – Right Superior Lobe (orange); Lobe 3 – Right Middle Lobe (yellow); Lobe 4 – Right Inferior Lobe (blue); Lobe 5 – Left Lung (green). PFU were determined via *M. smegmatis* plaque assay using a dilution of homogenates

We next addressed whether the pharmacokinetics, or retention of phage in the airways was disparate between aerosol or IV delivery as this may help to inform clinical practice regarding frequency and titer of dosing. In alignment with better dispersal across the lung lobes, phage delivered via aerosol were detectable through 6 h post-delivery (Fig. 2A, B), whereas IV delivery remained consistently low, variable, and near the assays’ limit of detection with no measurable reduction over time (Fig. 2A, C). These data suggest phages delivered via aerosol are cleared from the airway by 24 h post-delivery, but retain measurable PFU for at least 6 h and that clearance appears to occur equally across individual lung lobes (Fig. 2A, B).

**Fig. 3:**
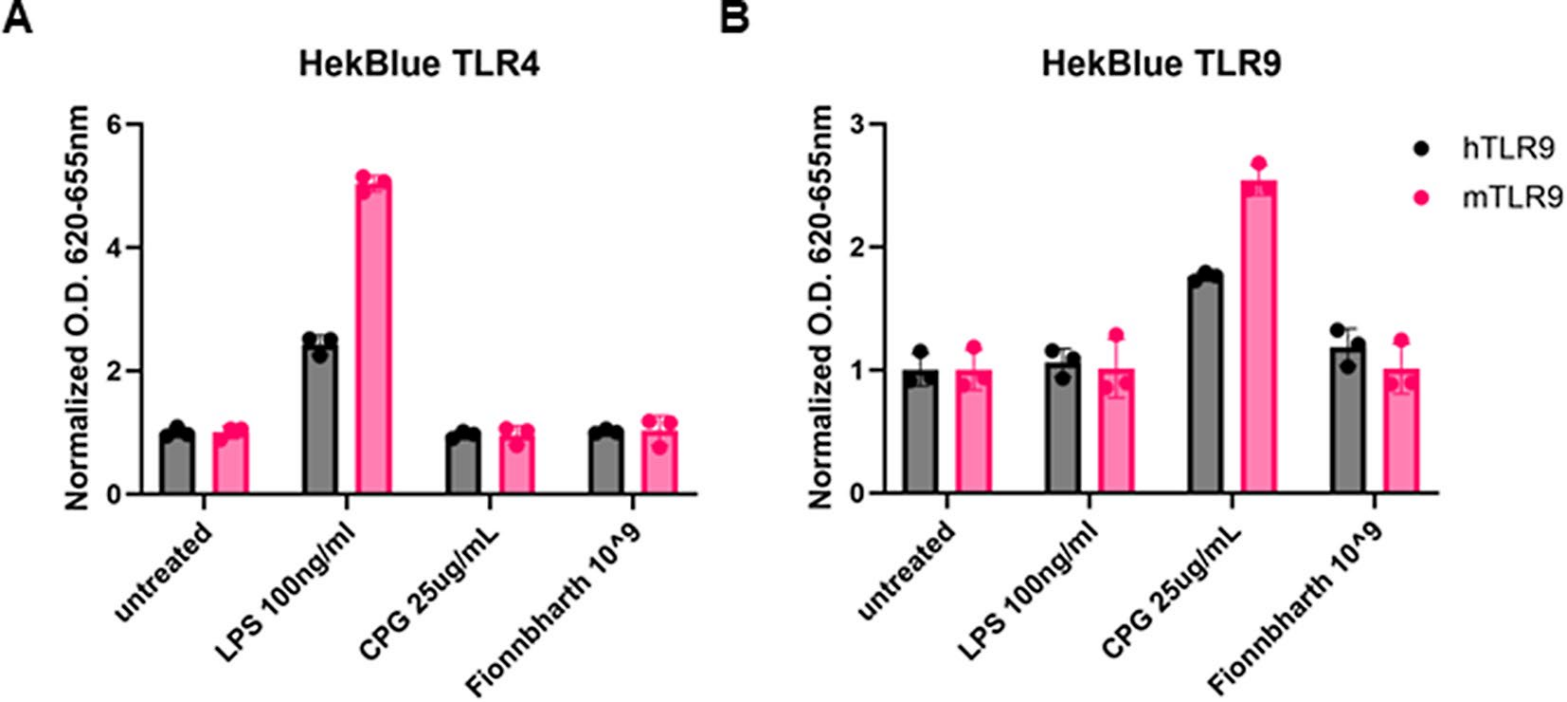
Innate immune response activation in HEK-Blue reporter cells. Activation of TLR4 (**A**) or TLR9 (**B**) in human (grey) and mouse (pink) TLR4 HEK-Blue reporter cell lines. LPS (lipopolysaccharide) was used as a TLR4 agonist control at 100 ng/mL, and CPG (cytosine guanosine dinucleotide) was used as a TLR9 agonist controls at 25 µg/mL. Fionnbharth (10^9^ PFU/mL) and controls were added to HEK-Blue cells in technical triplicates with detection media for detection of secreted embryonic alkaline phosphatase (SEAP). 24 h post-stimulation, plates were read at an optical density (O.D) of 620–655 nm and values were normalized to the average of the untreated wells

It remains unclear whether phages directly stimulate innate immune pathways [20]. Phage categorically could be recognized by host pathogen-associated molecular pattern (PAMP) receptors such as toll-like receptor (TLR) 9, which has been observed in phage-exacerbation of colitis in an ex vivo analysis [21]. Here we examined whether phage Fionnbharth would activate TLR9 or TLR4 receptors derived from mice or humans via HEK-Blue reporter cell lines. Highly purified phage did not activate human or mouse TLR4 (Fig. 3A) or TLR9 (Fig. 3B) innate immune responses compared to lipopolysaccharide (LPS) and cytosine guanosine dinucleotide (CPG) controls, respectively. This lack of antagonism of TLRs is a key assessment for potential therapeutic agents which may require repeated delivery.

Beyond innate immune activation, bacteriophages may contain epitopes that are recognized by the adaptive arms of the host immune system. Of the 32 Fionnbharth genes with functional predictions (GenBank, JN831653) seven are tail structural proteins and plausible targets for a neutralizing immune response; these were therefore selected for analysis using the Immune Epitope Database & Tools (IEDB) platform. The IEDB tool enables screening and prediction of probable immunogenic antigens across diverse human HLAs. The Fionnbharth Tail Terminator (gp18; GenBank ID AER26310.1), for example, contains two peptides with high MHC Class I binding scores (> 90%), 1 of which has high binding scores for three different HLA alleles. It also contained six peptides with high MHC Class II binding scores, one of which has high binding scores for two different HLA alleles. None of the peptides predicted to bind well to both MHC Class I and II were also identified as containing predicted linear B cell epitopes. Cumulatively, these data suggest that Fionnbharth may contain moderately immunogenic epitopes for specific HLAs (full summary in Supplemental Tables 1 and 2) and future work may be leveraged to reduce their immunogenicity with genetic engineering [22–24].

**Fig. 4:**
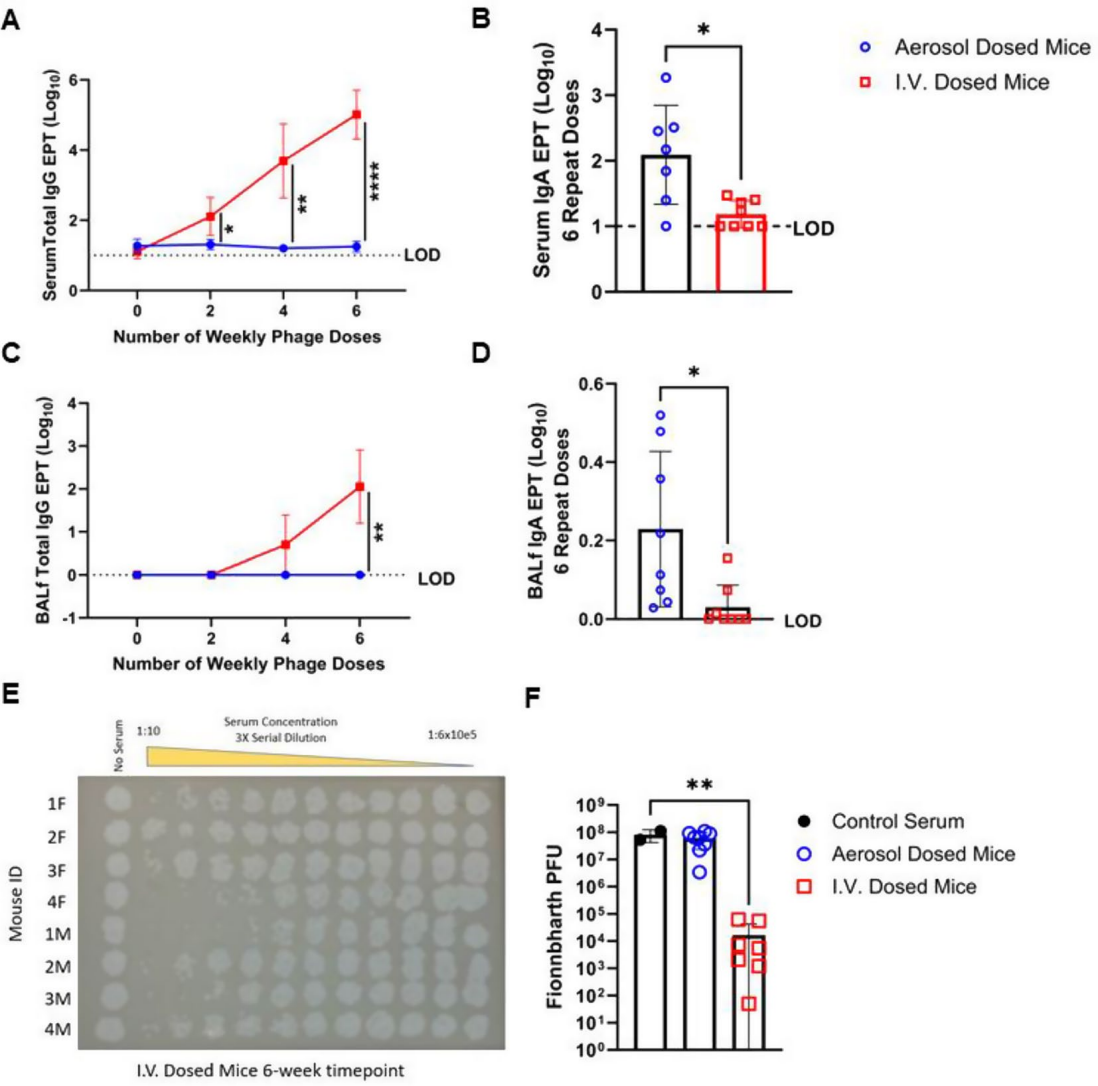
Antibody-mediated immune responses to weekly phage dosing. Fionnbharth was delivered via aerosol (open blue circles) or IV (open red squares) weekly for 6 weeks to a mixed cohort of male and female C57BL/6 mice. (**A**,** B**) Serum and (**C**,** D**) Bronchoalveolar lavage fluid (BALf) were collected every two weeks during treatment through week 7. Fionnbharth-specific Total IgG antibody responses in (**A**) Serum, and (**C**) BALf were measured by ELISA and reported as Log_10_ endpoint titers (EPT). Fionnbharth-specific IgA antibody responses were measured in (**B**) Serum, and (**D**) BALf by ELISA and reported as Log_10_ EPT. For the multiple timepoints of IgG, cohorts were compared via 2 way ANOVA and Šidáks correction, asterisks denote significant differences between delivery groups at the timepoint tested where **** *p* < 0.0001 and ** *p* < 0.01 and * *p* < 0.5. Aerosol and IV EPT at the single timepoints evaluated for IgA were compared using an unpaired t test with Welch correction Holm-Šídák method where * *p* ≤ 0.05. (**E**) Serum serially diluted from left to right across a representative plate to determine the lowest dilution of sample containing anti-phage antibodies that neutralized lytic activity against *M. smegmatis* via stamp neutralization assay, *n* = 4 Female (F) and *n* = 4 Male (M) mice shown from IV dosed cohort that received 6 doses of phage. (**F**) Quantified PFU from full plaque plate neutralization assay from control animals receiving no phage (closed black circle), aerosol (open blue circles) or IV (open red squares) dosed cohorts. Dosed cohorts (*n* = 7–8/group) compared to control using an ordinary One-way ANOVA and Dunnett’s multiple comparisons test where ** *p* = 0.0029

As a therapy for M.tb, bacteriophages will need to be repeatedly administered to patients experiencing TB disease, or to those exposed to infected individuals. Yet little is known about how the delivery route of phages may alter subsequent anti-phage host immune response magnitudes and kinetics. Reports describing individual cases of IV treatment for systemic nontuberculous mycobacterial infections have documented increasing anti-phage humoral responses over time with phage therapy [11, 25]. Here, we used the preclinical mouse model to test whether repeated weekly phage dosing for six weeks via aerosol would induce less anti-phage humoral responses compared to the same regimen with IV delivery. Consistent with clinical reports, mice receiving weekly phage via IV developed an escalating time-dependent anti-phage total IgG antibody response in both the serum and bronchoalveolar fluid (BALf) (Fig. 4A, C). Comparatively, aerosol phage delivery did not induce robust anti-phage total IgG in either tissue tested and remained at or near the lower limit of detection for the entirety of the six weeks of dosing (Fig. 4A, C). Aerosol phage delivery did, however, induce a moderate anti-phage IgA response in serum and BALf after six weeks of repeated dosing, whereas this response was significantly lower in IV treated cohorts (Fig. 4B, D). In addition to response magnitude, the functional capacity of the anti-phage responses was determined using a previously described neutralization assay [15]. We observed a 10^4^-fold reduction in Fionnbharth lytic activity after incubation with serum from mice that received six weekly IV doses of Fionnbharth compared to serum from untreated control mice (Fig. 4E, F). There was no observed reduction in lytic activity after incubation with serum from mice that received six weekly aerosol doses of Fionnbharth, despite the moderate Fionnbharth-specific IgA response detected (Fig. 4D, F).

**Fig. 5:**
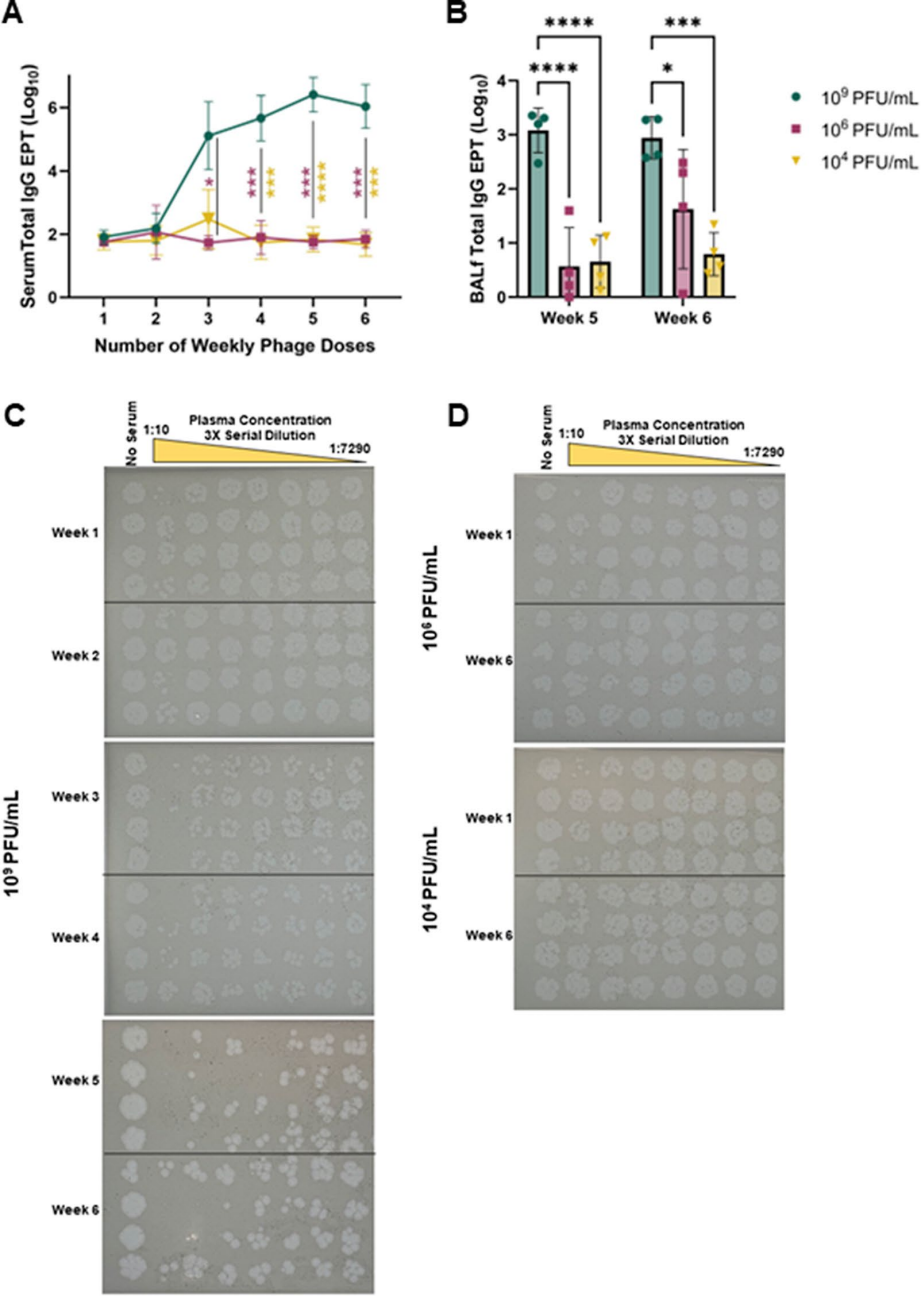
Immune responses to deescalating IV doses. C57BL/6 mice were given 10^9^ (green circles), 10^6^ (pink squares) or 10^4^ (yellow triangles) PFU/mL Fionnbharth phage weekly via IV for 6 consecutive weeks. (**A**) Serum was collected weekly and total IgG antibody responses were measured by ELISA. De-escalation cohorts were compared to the high dose 10^9^ PFU/mL overtime using a 2 way ANOVA and Dunnett’s multiple comparison test, where **** *p* < 0.0001, *** *p* < 0.001, and * *p* = 0.0114. (**B**) BALf was collected at weeks 5 and 6 and total IgG antibody responses were measured by ELISA. De-escalation cohorts were compared to the high dose 10^9^ PFU/mL at 5 and 6 week timepoints using 2 way ANOVA with Šidáks correction, asterisks denote significant differences between delivery groups where **** *p* < 0.0001, *** *p* = 0.003, and *p = 0.0183. (**C**) Stamp plate neutralization of weekly serum samples from high dose (1 × 10^9^ PFU/mL) IV delivery group. (**D**) Stamp plate neutralization of weeks 1 and 6 serum samples from medium (upper, 1 × 10^6^ PFU/mL) and low (lower, 1 × 10^4^ PFU/mL) IV delivery groups

Interestingly, when the dose of IV phage is reduced, the corresponding anti-phage immune response is comparatively dampened in the preclinical mouse model of repeated dosing. In this de-escalating IV delivery study, a serum Fionnbharth-specific total IgG response was detected only in the high dose group (10^9^ PFU/mL) (Fig. 5A), and the response magnitude was consistent with the previous aerosol vs. IV delivery experiment. For the high dose group (10^9^ PFU/mL), total IgG EPT was significantly higher at four weeks compared to the one week baseline. We did not detect a significant increase in total IgG EPTs compared to the week one baseline from either of the lower dose groups (10^6^, 10^4^ PFU/mL, Fig. 5A). After 3 weekly doses, the total IgG EPT was significantly higher in the highest dose group compared to the medium dose group and remained significant out to six weekly doses. After four weekly doses, the total IgG EPT was significantly higher in the highest dose group compared to the low dose group and remained significant out to six weekly doses. In BALf, at the five-week timepoint Fionnbharth-specific total IgG responses were detected only in the high dose group, but after six weeks a total IgG response was detected in the middle dose group as well (10^6^ PFU/mL, Fig. 5B). Antibody-mediated neutralization was characterized using the stamp neutralization assay. In the high dose group (10^9^ PFU/mL), consistent with the previous weekly IV dosing experiment with the same dose, there was an increase in Fionnbharth neutralization after each weekly dose (Fig. 5C). In the middle and low dose groups there was no observed increase in neutralization between the week one baseline and after six weekly doses (Fig. 5D). While these results suggest a dose de-escalation for the IV route of delivery may mitigate the neutralizing anti-phage immunity induced by the recipient, it does not address the accompanying low pulmonary deposition observed in the high dose IV group (Fig. 1).

**Fig. 6:**
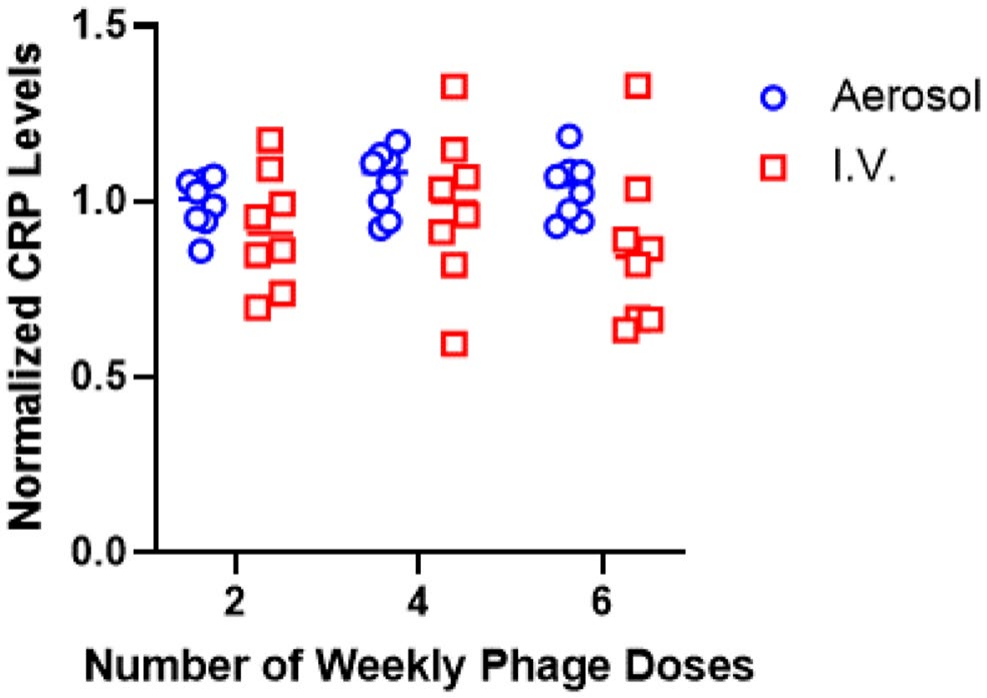
CRP quantification in serum from mice dosed with Fionnbharth weekly via aerosol or I.V routes. Fionnbharth was delivered via aerosol (open blue circles) or IV (open red squares) weekly for 6 weeks to a mixed cohort of male and female C57BL/6 mice. Serum samples were evaluated for CRP using a commercial kit and normalized to untreated controls without phage exposure. Aerosol and IV cohorts were compared over time with a 2-way ANOVA and Šidáks (between groups) or Dunnett’s (within a group) multiple comparison test, no significant differences between groups or within a group over time were observed

In addition to ex vivo immunogenicity and predictive epitope mapping, C-reactive Protein (CRP) was used as a general marker of induced inflammation from in vivo repeated delivery studies. Serum from aerosol or IV delivery cohorts were collected after 2, 4 and 6 doses and evaluated for CRP. There was no significant difference between cohorts or within a cohort over time (Fig. 6), suggesting phage delivery by either route is generally well tolerated in this preclinical model, which mimics clinical observations for aerosol and IV phage delivery [11].

**Fig. 7:**
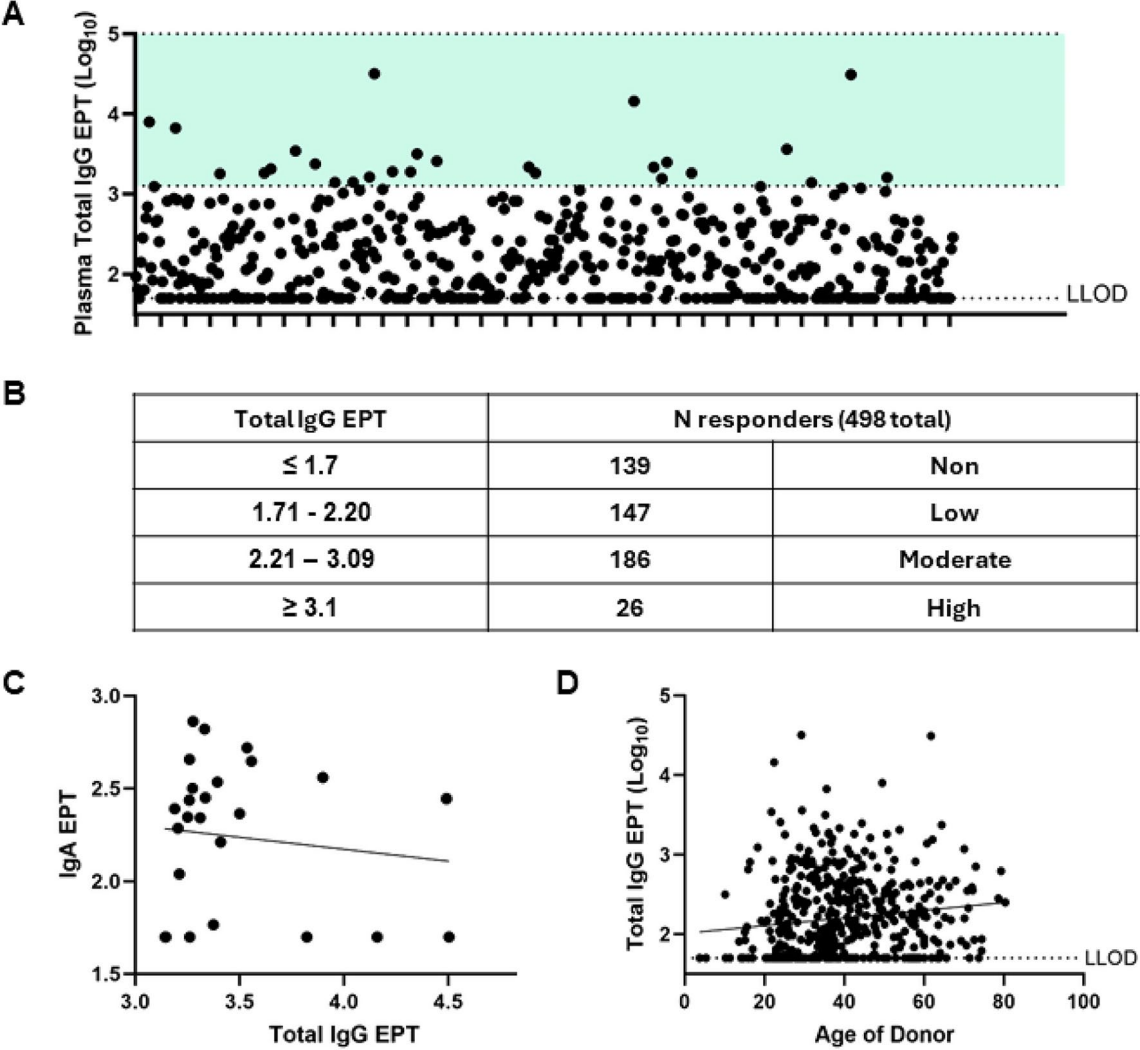
Human background Fionnbharth-specific humoral immune responses. Plasma from 498 healthy donors evaluated for anti-Fionnbharth total IgG responses by ELISA. (**A**) Graphical and (**B**) numerical representation of Log_10_ Total IgG EPT values. (**C**) High responder donors (> 3.1 total IgG EPT, *n* = 26 total) and sample matched anti-Fionnbharth IgA responses evaluated for correlation using a simple linear regression where R^2^ = 0.016 and *p* = 0.543. (**D**) Donor age and anti-Fionnbharth Total IgG responses evaluated for correlation using a simple linear regression where R^2^ = 0.018 and *p* = 0.0031 (*n* = 484 donors reporting age data)

The background anti-phage immunity in laboratory mice should be low or near zero, but given many phages are isolated from environmental samples, humans may have historical exposures and harbor some background immunity. Using an anti-Fionnbharth ELISA and human donor plasma from a prospective study in the Seattle area, we evaluated EPT responses in samples from 498 individuals. As expected, we observed a relatively low responder rate where, 139/498 (27.9%) had no response, 333/498 (66.9%) had a low or moderate Fionnbharth-specific total IgG response, and only 26/498 (5.2%) had a high response (EPT categories described in Fig. 7A, B). The 26 high responders had variable anti-Fionnbharth IgA responses, with no association by simple linear regression between total IgG and IgA EPT (R^2^ = 0.016, *p* = 0.543, Fig. 7C). High responders were also evaluated for Fionnbharth neutralization by stamp neutralization assay, and none of the 26 plasma samples neutralized phage after co-incubation (data not shown). Additionally, in donors that reported age data at the time of sample collection (484/498) we observed a weak but significant correlation between age and anti-Fionnbharth Total IgG EPT (R^2^ = 0.018, *p* = 0031, Fig. 7D) by simple linear regression.

## Discussion

The results described here substantially advance the prospects of using mycobacteriophages as prophylaxis and therapy against *M. tuberculosis* infections. This includes relatively low predicted epitope immunogenicity, no induction of overtly inflammatory molecules like CRP, and low background phage-specific antibody frequency rate in healthy human donors. This work includes a head-to-head evaluation of aerosol versus IV phage dosing in a mouse model to systematically interrogate the dispersal, lung coverage, and subsequent host anti-phage immune responses generated. Prior studies vary greatly regarding pharmacokinetic clearance of phage after IV delivery (~ 30 min to > 8 h), but generally there is a rapid systemic dissemination followed by clearance from the blood [26]. The relatively poor delivery of phage particles to the lungs using IV delivery shown here is in alignment with previously reported rapid disbursement. Preclinical studies suggest that the spleen and liver accumulate the highest concentration of phage after IV delivery which may explain why there is such rapid elimination and poor distribution to other organs, including the lung, using this route [26]. However, IV phage delivery has produced favorable outcomes against pulmonary infections [11], so it remains to be seen how phage dosage influences effective reduction of pulmonary M.tb bacilli and whether IV phage delivery can reach those thresholds clinically.

Pulmonary localization or accumulation seems route-dependent in previous reports [27], which is consistent with our findings where aerosol delivery enhanced the biodistribution of phage across all lung lobes. Phage localization and persistence experiments were performed using non-perfused lung homogenates that capture both airway phage and any phage in the pulmonary circulation, and thus it is likely that the small recovery of pulmonary phage after IV dosing is an overestimation of what localizes to the airway. Fionnbharth dosed via aerosol at 7.5 × 10^10^ PFU/mL in the nebulizer persists in mouse lungs for six hours. If this dynamic translates well to a human airway and phage pharmacokinetics, then this type of treatment may provide a protective effect over the course of a work shift, or overnight for household contacts of active TB disease cases. In this preclinical model, the aerosol dosing method was both more effective at delivering phage to the lungs and did not elicit a neutralizing antibody response. Conversely, IV dosing resulted in reduced dissemination to the pulmonary space and elicited high titers of anti-phage neutralizing antibodies, similar to what has been observed clinically in some cases [11, 28]. Neutralization in clinical cases can correspond with reduced efficacy of phage therapy but has not been observed in all cases [11, 15]. Despite observing decreased neutralization with de-escalating IV phage doses, lowering phage titer may further decrease deposition to the target organ which may limit meaningful protection.

When considering CRP as a marker of overt inflammatory responses, repeated phage dosing by either route appeared well tolerated regardless of the exposure method, likely due to the purity of the preparations. Despite observations of unrelated phages activating innate immune responses in a variety of contexts [29], Fionnbharth did not activate TLR4 or TLR9 innate immune response pathways in these studies. Pairing reporter cell lines with the predicted immunogenicity pipeline performed here serves as a model workflow for evaluating the potential intrinsic immune stimulation encoded in each phage genome, which can be further confirmed in experimental in vivo or ex vivo studies. The predicted immunogenicity of the selected Fionnbharth tail proteins in humans was overall quite low, but we observed a strong and highly neutralizing immune response in mice with repeated IV dosing. This may be due to epitopes in other structural proteins not evaluated, or a factor of the repeated exposures. Fionnbharth was isolated in La Jolla, California under the Science Education Alliance-Phage Hunters Advancing Genomics and Evolutionary Science (SEA-PHAGES) program (as described in PhagesDB.org entry [30]) from an environmental sample. We observed a measurable anti-Fionnbharth response in over half of the normal healthy donor plasma isolated from a Seattle-based prospective cohort study [19], albeit a majority were low to moderate responders. High responders in this population did not demonstrate subsequent neutralization of Fionnbharth, suggesting this baseline response would not negate therapeutic use, and may have been generated against other structural proteins in Fionnbharth or related environmental phages [31]. Global populations have unique exposures to pathogens and presumably bacteriophages so it remains to be seen whether regions with endemic TB, which would benefit most from phage therapy, would have similar baseline magnitude and non-neutralizing anti-phage antibody responses.

## Conclusion

Aerosol delivery is a viable option for repeat dosing of phages and should be prioritized for further efficacy evaluation. This can be leveraged not only for postexposure prophylaxis approaches but also therapeutic strategies for TB and likely other pulmonary infections. In selecting phages for clinical application, the IEDB platform can help inform phage selection as well as providing a starting point for high-throughput screening of protein candidates for immunogenicity reduction, especially in cases where aerosol delivery is not advised. Future work will explore increasing the concentration of phage administered via aerosol to increase the persistence of phage in the lungs. Beyond the host-phage interaction and route of delivery explored here, the most critical endpoints will be preclinical and clinical efficacy studies using prioritized bacteriophage cocktails [13, 32, 33] for the prevention and treatment of M.tb infections.

## Electronic Supplementary Material

Below is the link to the electronic supplementary material.

Supplementary Material 1

## Data Availability

No datasets were generated or analysed during the current study.

## Abbreviations

TB: Tuberculosis
M.tb: Mycobacterium tuberculosis
Phage: Mycobacteriophage
IV: Intravenous
Fionnbharth: FionnbharthΔ45Δ47
BALf: Bronchoalveolar Lavage fluid
TLR: Toll Like Receptor
CDC: Center for Disease Control
BCG: Mycobacterium bovis bacille Calmette- Guérin
Phage: Bacteriophage
NTM: Nontuberculous mycobacteria
CFU: Colony forming units
SCRI: Seattle Children’s Research Institute
LDA: Low-dose aerosol
PFU: Plaque Forming Units
MBTA: Middlebrook Top Agar
CRP: C-Reactive Protein
O.D: Optical density
IEDB: Immune Epitope Database & Tools
MHC: Major histocompatibility complex
HRP: Horseradish peroxidase
TMB: Tetramethylbenzidine
EPT: End Point Titer
LPS: Lipopolysaccharide
CPG: Cytosine guanosine dinucleotide
