# Supplementary material for: Evaluation of host immune responses to Mycobacteriophage Fionnbharth by route of delivery
Source: Virol J. 2025 Jan 20;22:14. doi: 10.1186/s12985-024-02552-2 (PMC11748884; doi:10.1186/s12985-024-02552-2)
Supplement: Supplementary file 1 — Supplementary Material 1 [file 12985_2024_2552_MOESM1_ESM.docx]

**Evaluation of Host Immune Responses to Mycobacteriophage Fionnbharth by Route of Delivery**

Thomas Smytheman^1^, Tiffany Pecor^1^, Dana E. Miller^1^, Debora Ferede^1^, Suhavi Kaur^1^, Matthew H. Harband^1^, Hazem F. M. Abdelaal^1^, Carlos A. Guerrero-Bustamante^2^, Krista G. Freeman^2^, Whitney E. Harrington^1,3^, Lisa M. Frenkel^1,2^, Graham F. Hatfull^2^, Rhea N. Coler^1,3,4*^, Sasha E. Larsen^1^

^1^Seattle Children’s Research Institute, Center for Global Infectious Disease Research, Seattle, WA.

^2^Department of Biological Services, University of Pittsburgh, Pittsburgh, PA.

^3^Department of Pediatrics, University of Washington School of Medicine, Seattle, WA, USA.

^4^Department of Global Health, University of Washington, Seattle, WA, USA.

**Supplementary Tables**

| **Supplementary Table 1. Predicted MHC Class I and overlapping B cell epitopes in Fionnbharth tail proteins** | | | | |
| --- | --- | --- | --- | --- |
| **Gene** | **Peptide** | **Allele** | **Immunogenicity Score** | **Overlapping Predicted B cell epitope** |
| Gene 18  Tail Terminator | YLADYLIRV | HLA-A*02:01 | 0.16354 |  |
|  | YLADYLIRV | HLA-A*02:03 | 0.16354 |  |
|  | YLADYLIRV | HLA-A*02:06 | 0.16354 |  |
|  | RINGTKHHY | HLA-A*30:02 | -0.1143 |  |
| Gene 19  Major Tail Protein | YEVPLFQVL | HLA-B*40:01 | 0.028 |  |
|  | MTFTPTFKV | HLA-A*68:02 | 0.0756 |  |
|  | EPMTFTPTF | HLA-B*35:01 | 0.14592 |  |
|  | EPMTFTPTF | HLA-B*53:01 | 0.14592 |  |
|  | **SWGDPTQLF** | HLA-A*24:02 | 0.05258 | DV**SWGDPTQLF**AASPSDL |
|  | **DETVPIEVW** | HLA-B*44:03 | 0.26464 | GVTVTIN**DETVPIEVW**GGDEVGQLRDS |
|  | **DETVPIEVW** | HLA-B*44:02 | 0.26464 | GVTVTIN**DETVPIEVW**GGDEVGQLRDS |
|  | SVYEDKMIR | HLA-A*68:01 | -0.15229 |  |
|  | LPKRCSLVL | HLA-B*07:02 | -0.21139 |  |
| Gene 22  Tape Measure Protein | RTSASIQAW | HLA-B*58:01 | -0.12505 |  |
|  | RTSASIQAW | HLA-B*57:01 | -0.12505 |  |
|  | LPNGVNFEM | HLA-B*35:01 | 0.22341 |  |
|  | KTETGRKLW | HLA-B*57:01 | -0.0352 |  |
|  | VGSTISWLW | HLA-B*57:01 | 0.13969 |  |
|  | TVASTVAEK | HLA-A*68:01 | 0.01441 |  |
|  | GPADVLSAF | HLA-B*35:01 | -0.05198 |  |
|  | FLAGVPDSV | HLA-A*02:03 | -0.00138 |  |
|  | FLAGVPDSV | HLA-A*02:01 | -0.00138 |  |
|  | TLKNGVVAV | HLA-A*02:03 | 0.05305 |  |
|  | VGSTISWLW | HLA-B*58:01 | 0.13969 |  |
|  | **ESNGAEVGR** | HLA-A*68:01 | 0.21899 | I**ESNGAE** |
|  | KLVGSTISW | HLA-A*32:01 | -0.0614 |  |
|  | TLTPIINLW | HLA-B*58:01 | 0.24438 |  |
|  | RVIDWFVRL | HLA-A*02:06 | 0.4565 |  |
|  | TLTPIINLW | HLA-B*57:01 | 0.24438 |  |
|  | MLSPLSSEV | HLA-A*02:03 | -0.31251 |  |
|  | RLKDTIAGF | HLA-B*15:01 | 0.16822 |  |
|  | KLAPGIRKA | HLA-A*02:03 | 0.0775 |  |
|  | ALKTAIQFV | HLA-A*02:03 | 0.10308 |  |
|  | KVDAVTFRK | HLA-A*11:01 | 0.25235 |  |
|  | SLLGYPAMV | HLA-A*02:01 | -0.05312 |  |
|  | SLFSNPFEW | HLA-A*32:01 | 0.01209 |  |
|  | AEKAKAVAL | HLA-B*40:01 | -0.1461 |  |
|  | TVASTVAEK | HLA-A*11:01 | 0.01441 |  |
|  | KTETGRKLW | HLA-B*58:01 | -0.0352 |  |
|  | NLWNSFFQV | HLA-A*02:01 | 0.04561 |  |
|  | TELTEYLKL | HLA-B*40:01 | -0.00588 |  |
|  | MLSPLSSEV | HLA-A*02:01 | -0.31251 |  |
|  | MVIPFATAF | HLA-B*35:01 | 0.23849 |  |
|  | KLVGSTISW | HLA-B*58:01 | -0.0614 |  |
|  | KLVGSTISW | HLA-B*57:01 | -0.0614 |  |
|  | RTSASIQAW | HLA-A*32:01 | -0.12505 |  |
|  | VVALGTALW | HLA-B*58:01 | 0.09762 |  |
|  | LGNAIKTVW | HLA-B*57:01 | 0.02075 |  |
|  | FLAGVPDSV | HLA-A*02:06 | -0.00138 |  |
|  | VVALGTALW | HLA-B*57:01 | 0.09762 |  |
|  | VPAFQGIGL | HLA-B*07:02 | 0.18172 |  |
| Gene 23  Minor Tail Protein | RSYPKFLVK | HLA-A*03:01 | -0.0974 |  |
|  | FTRTHGWRW | HLA-B*57:01 | 0.33644 |  |
|  | GSFTRTHGW | HLA-B*57:01 | 0.2111 |  |
|  | SAWRIFDSW | HLA-B*57:01 | 0.28584 |  |
|  | RSYPKFLVK | HLA-A*30:01 | -0.0962 |  |
|  | AESDGSFEM | HLA-B*40:01 | 0.00319 |  |
|  | YPFFAKRAL | HLA-B*07:02 | 0.05744 |  |
|  | QMNPTHPHW | HLA-B*57:01 | 0.06453 |  |
|  | RMIPLPKLY | HLA-A*30:02 | 0.00432 |  |
|  | SAWRIFDSW | HLA-B*58:01 | 0.28584 |  |
|  | QMNPTHPHW | HLA-B*58:01 | 0.06453 |  |
|  | RMIPLPKLY | HLA-B*15:01 | -0.17768 |  |
|  | PVDSQVYKY | HLA-A*01:01 | -0.36953 |  |
|  | KPSKTTLSV | HLA-B*07:02 | -0.30238 |  |
|  | RSYPKFLVK | HLA-A*11:01 | -0.0974 |  |
|  | GSFTRTHGW | HLA-B*58:01 | 0.2111 |  |
|  | YVVGSIRER | HLA-A*68:01 | 0.11386 |  |
|  | MPQHYKMAW | HLA-B*53:01 | -0.33699 |  |
|  | KYLRNSQLL | HLA-A*24:02 | -0.12003 |  |
| Gene 24  Minor Tail Protein | FVTETIYQV | HLA-A*02:01 | 0.20563 |  |
|  | **FMDPAVQPI** | HLA-A*02:01 | -0.03124 | **FMDPAVQ** |
|  | ILKPPTIGV | HLA-A*02:03 | 0.0767 |  |
|  | FVTETIYQV | HLA-A*02:06 | 0.20563 |  |
|  | KTRPYQAFK | HLA-A*03:01 | -0.00558 |  |
|  | SMFRSKAQK | HLA-A*03:01 | -0.30868 |  |
|  | RLWDKNMQY | HLA-A*03:01 | -0.33775 |  |
|  | KTRPYQAFK | HLA-A*30:01 | -0.02238 |  |
|  | RLWDKNMQY | HLA-A*30:02 | -0.18955 |  |
|  | KVVNVRVKR | HLA-A*31:01 | 0.00465 |  |
|  | RLWDKNMQY | HLA-A*32:01 | -0.33775 |  |
|  | TVSSGLTLR | HLA-A*68:01 | -0.17133 |  |
|  | **ITIDPYPNR** | HLA-A*68:01 | 0.0381 | **DPYPNR**RN |
|  | TTGFINLAR | HLA-A*68:01 | 0.26581 |  |
|  | FVTETIYQV | HLA-A*68:02 | 0.20563 |  |
|  | APALPKITF | HLA-B*07:02 | -0.07726 |  |
|  | **APDPVKAPL** | HLA-B*07:02 | -0.14022 | TIGVDG**APDPVKAP** |
|  | SPHPELAAI | HLA-B*07:02 | 0.14228 |  |
|  | RLWDKNMQY | HLA-B*15:01 | -0.33775 |  |
|  | DAHSVTEAM | HLA-B*35:01 | 0.02813 |  |
|  | NPLNWPVQM | HLA-B*35:01 | 0.16231 |  |
|  | GEASNVLNL | HLA-B*40:01 | -0.13435 |  |
|  | REHEHSAII | HLA-B*40:01 | 0.0978 |  |
|  | YPNRRNWRW | HLA-B*53:01 | 0.31147 |  |
|  | KARPLIRLW | HLA-B*57:01 | 0.15732 |  |
|  | VTRTAQAFW | HLA-B*57:01 | 0.08634 |  |
|  | LTGGKSPGW | HLA-B*57:01 | -0.31019 |  |
|  | KARPLIRLW | HLA-B*58:01 | 0.15732 |  |
|  | VTRTAQAFW | HLA-B*58:01 | 0.08634 |  |
| Gene 26  Minor Tail Protein | AQRQPILSY | HLA-B*15:01 | -0.0913 |  |
|  | SLNPHFHLR | HLA-A*31:01 | 0.14926 |  |
|  | **DESDKGKAW** | HLA-B*44:03 | -0.31492 | TL**DESDKGK** |
|  | **DESDKGKAW** | HLA-B*44:02 | -0.31492 | TL**DESDKGK** |
|  | RGFGNISSW | HLA-B*57:01 | -0.0452 |  |
|  | RGFGNISSW | HLA-B*58:01 | -0.0452 |  |
|  | SWTTIQPHF | HLA-A*24:02 | 0.09112 |  |
|  | AQRQPILSY | HLA-A*30:02 | -0.08194 |  |
| Gene 28  Minor Tail Protein | **SLADPTEQI** | HLA-A*02:01 | 0.07758 | GRQTDDDV**SLADPTEQI** |
|  | **SLADPTEQI** | HLA-A*02:03 | 0.07758 | GRQTDDDV**SLADPTEQI** |
|  | HIIDRIVNL | HLA-A*02:03 | 0.27226 |  |
|  | HIIDRIVNL | HLA-A*02:06 | 0.27226 |  |
|  | SLYEPGIPK | HLA-A*03:01 | 0.22649 |  |
|  | SLYEPGIPK | HLA-A*11:01 | 0.22649 |  |
|  | NVFDSIAYK | HLA-A*11:01 | 0.05536 |  |
|  | VYGTTMRVF | HLA-A*24:02 | -0.05332 |  |
|  | NVFDSIAYK | HLA-A*68:01 | 0.05536 |  |
|  | TTYADRFGR | HLA-A*68:01 | 0.22709 |  |
|  | TAVYGTTMR | HLA-A*68:01 | 0.00938 |  |
|  | STNTGTFVR | HLA-A*68:01 | 0.22942 |  |
|  | DTAGITDGV | HLA-A*68:02 | 0.25146 |  |
|  | ETINVVGKL | HLA-A*68:02 | 0.01835 |  |
|  | NTSGVSMPV | HLA-A*68:02 | -0.28981 |  |
|  | SPAKFVESL | HLA-B*07:02 | -0.0636 |  |
|  | IPKLPELKL | HLA-B*07:02 | -0.13307 |  |
|  | VLYKNGVVY | HLA-B*15:01 | -0.13364 |  |
|  | NPATNTVTF | HLA-B*35:01 | 0.13952 |  |
|  | GEAPLPFIL | HLA-B*40:01 | 0.15686 |  |
|  | VESLGSLIL | HLA-B*40:01 | -0.11919 |  |
|  | GELAARLGL | HLA-B*40:01 | 0.13303 |  |
|  | GEDFRRWGW | HLA-B*44:02 | 0.42366 |  |
|  | VPIDRNPLW | HLA-B*53:01 | 0.09399 |  |
|  | NPATNTVTF | HLA-B*53:01 | 0.13952 |  |
|  | IVSHLQQTW | HLA-B*57:01 | -0.21607 |  |
|  | TSNGHDAVW | HLA-B*57:01 | 0.14152 |  |
|  | IVSHLQQTW | HLA-B*58:01 | -0.21607 |  |
|  | TSNGHDAVW | HLA-B*58:01 | 0.14152 |  |
|  | VTSSTLDDW | HLA-B*58:01 | -0.16113 |  |

| **Supplementary Table 2. Predicted MHC Class II and overlapping B cell epitopes in Fionnbharth tail proteins** | | | | |
| --- | --- | --- | --- | --- |
| **Gene** | **peptide** | **allele** | **Immunogenicity score** | **Overlapping Predicted B cell epitope** |
| Gene 19  Major Tail Protein | TPTFKVLKNTDGNHV | HLA-DRB1*04:01 | 0.9368 |  |
|  | FTPTFKVLKNTDGNH | HLA-DRB1*04:01 | 0.9307 |  |
|  | TPTFKVLKNTDGNHV | HLA-DRB1*01:01 | 0.9293 |  |
|  | NEPMTFTPTFKVLKN | HLA-DRB1*07:01 | 0.922 |  |
|  | FTPTFKVLKNTDGNH | HLA-DRB1*01:01 | 0.9208 |  |
|  | GNRMRVLINNKLPKR | HLA-DRB1*15:01 | 0.9096 |  |
|  | HNEPMTFTPTFKVLK | HLA-DRB1*07:01 | 0.9023 |  |
| Gene 22  Tape Measure Protein | AVTFRKVIAENIGGA | HLA-DRB1*07:01 | 0.9779 |  |
|  | **NRVYNAQNSVRQAEQ** | HLA-DRB1*04:01 | 0.977 | **NSVRQAEQ**SVDDRQYAVD |
|  | DAVTFRKVIAENIGG | HLA-DRB1*07:01 | 0.9744 |  |
|  | AVTFRKVIAENIGGA | HLA-DRB1*01:01 | 0.9728 |  |
|  | SNRVYNAQNSVRQAE | HLA-DRB1*04:01 | 0.9691 |  |
|  | TAAQWAFNAALRANP | HLA-DRB3*02:02 | 0.9657 |  |
|  | AAQWAFNAALRANPI | HLA-DRB3*02:02 | 0.9628 |  |
|  | PEDFHSGTPQLAPGQ | HLA-DQA1*05:01/DQB1*03:01 | 0.9569 |  |
|  | DAVTFRKVIAENIGG | HLA-DRB1*01:01 | 0.9564 |  |
|  | VDAVTFRKVIAENIG | HLA-DRB1*07:01 | 0.9511 |  |
|  | VPEDFHSGTPQLAPG | HLA-DQA1*05:01/DQB1*03:01 | 0.949 |  |
|  | GKAFTDDLNMLSDRG | HLA-DRB1*04:01 | 0.9384 |  |
|  | KAPLRALGSFLAGVP | HLA-DRB1*01:01 | 0.9356 |  |
|  | **VSAIANYATGRAPFS** | HLA-DRB1*15:01 | 0.9317 | **ATGRAPF** |
|  | SSNRVYNAQNSVRQA | HLA-DRB1*04:01 | 0.9316 |  |
|  | EDFHSGTPQLAPGQY | HLA-DQA1*05:01/DQB1*03:01 | 0.9293 |  |
|  | EVPEDFHSGTPQLAP | HLA-DQA1*05:01/DQB1*03:01 | 0.9285 |  |
|  | VTFRKVIAENIGGAA | HLA-DRB1*07:01 | 0.9263 |  |
|  | **NGGPAGGHTAATLPN** | HLA-DQA1*05:01/DQB1*03:01 | 0.9241 | Y**NGGPAGGHTAATLP** |
|  | RVYNAQNSVRQAEQS | HLA-DRB1*04:01 | 0.9231 |  |
|  | **LGGLFKGAGDQAADE** | HLA-DRB1*04:01 | 0.9191 | **GAGDQAADE**LGAATAPGGFAAGAADA |
|  | **GGLFKGAGDQAADEL** | HLA-DRB1*04:01 | 0.9124 | **GAGDQAADE**LGAATAPGGFAAGAADA |
|  | AVSAIANYATGRAPF | HLA-DRB1*15:01 | 0.9114 |  |
|  | **PASLRNEIRAEQTQR** | HLA-DRB1*11:01 | 0.9071 | **RAEQTQR**TRT |
|  | PEHFTGLDGLTGSTY | HLA-DRB1*01:01 | 0.9039 |  |
|  | VTAAQWAFNAALRAN | HLA-DRB3*02:02 | 0.9031 |  |
|  | YNGGPAGGHTAATLP | HLA-DQA1*05:01/DQB1*03:01 | 0.9024 |  |
|  | SGKAFTDDLNMLSDR | HLA-DRB1*04:01 | 0.9021 |  |
|  | GNGQYGGQAAGADDP | HLA-DQA1*05:01/DQB1*03:01 | 0.9018 |  |
|  | DRQYAVDKAQKRLDE | HLA-DRB3*01:01 | 0.9007 |  |
| Gene 23  Minor Tail Protein | EGRMIPLPKLYAEDG | HLA-DRB1*11:01 | 0.9675 |  |
|  | VEGRMIPLPKLYAED | HLA-DRB1*11:01 | 0.9579 |  |
|  | **RKTIVTEKDPVDSQV** | HLA-DRB1*04:05 | 0.932 | TDPT**RKTIVTEKDPVDS** |
|  | DSQVYKYLRNSQLLN | HLA-DRB1*01:01 | 0.9225 |  |
|  | **TRKTIVTEKDPVDSQ** | HLA-DRB1*04:05 | 0.922 | TDP**TRKTIVTEKDPVDS** |
|  | EGRMIPLPKLYAEDG | HLA-DRB1*08:02 | 0.9168 |  |
|  | SQVYKYLRNSQLLNI | HLA-DRB1*01:01 | 0.9151 |  |
|  | GRMIPLPKLYAEDGA | HLA-DRB1*11:01 | 0.9111 |  |
|  | DGSFEMNMSIDAPYP | HLA-DRB3*02:02 | 0.9078 |  |
|  | DGAYMMVDTDPTRKT | HLA-DRB1*04:01 | 0.9055 |  |
|  | GVEGRMIPLPKLYAE | HLA-DRB1*11:01 | 0.9012 |  |
| Gene 24  Minor Tail Protein | ETIYQVIGTNKVIDP | HLA-DRB1*01:01 | 0.9761 |  |
|  | ETIYQVIGTNKVIDP | HLA-DRB1*07:01 | 0.9687 |  |
|  | HPELAAIVGEKLARP | HLA-DRB1*01:01 | 0.9683 |  |
|  | TETIYQVIGTNKVID | HLA-DRB1*07:01 | 0.9494 |  |
|  | GTAYTVSSGLTLREG | HLA-DRB1*07:01 | 0.9432 |  |
|  | ETIYQVIGTNKVIDP | HLA-DRB1*04:01 | 0.9424 |  |
|  | PHPELAAIVGEKLAR | HLA-DRB1*01:01 | 0.942 |  |
|  | TETIYQVIGTNKVID | HLA-DRB1*01:01 | 0.9379 |  |
|  | DPAVQPIKAWLLPGN | HLA-DRB1*01:01 | 0.9338 |  |
|  | **SESPIAQVTRTAQAF** | HLA-DRB1*11:01 | 0.9335 | GDD**SESESPIAQ** |
|  | GGTAYTVSSGLTLRE | HLA-DRB1*07:01 | 0.9279 |  |
|  | ESPIAQVTRTAQAFW | HLA-DRB1*11:01 | 0.9212 |  |
|  | **NVRVKRDENGTRTVV** | HLA-DRB1*03:01 | 0.9095 | **KRDENGT** |
|  | TETIYQVIGTNKVID | HLA-DRB1*04:01 | 0.9071 |  |
|  | ETIYQVIGTNKVIDP | HLA-DRB1*04:05 | 0.9065 |  |
|  | VNVRVKRDENGTRTV | HLA-DRB1*03:01 | 0.9012 |  |
|  | LARNYWPLLALPTNA | HLA-DRB1*01:01 | 0.9007 |  |
|  | SGPYGYLEHFEQGGG | HLA-DRB1*11:01 | 0.9001 |  |
| Gene 26  Minor Tail Protein | GSDFVAVQPGPAGPP | HLA-DRB1*01:01 | 0.9854 |  |
|  | TGSDFVAVQPGPAGP | HLA-DRB1*01:01 | 0.9722 |  |
|  | ASELIPIKDTLDESD | HLA-DRB1*04:05 | 0.9572 |  |
|  | SASELIPIKDTLDES | HLA-DRB1*04:05 | 0.9438 |  |
|  | GSDFVAVQPGPAGPP | HLA-DRB1*08:02 | 0.9435 |  |
|  | **WYGIVADKDTPGGFA** | HLA-DRB1*04:01 | 0.9404 | **ADKDTPGGF** |
|  | SDFVAVQPGPAGPPG | HLA-DRB1*01:01 | 0.9396 |  |
|  | GSDFVAVQPGPAGPP | HLA-DRB1*11:01 | 0.922 |  |
|  | **GSDFVAVQPGPAGPP** | HLA-DRB1*09:01 | 0.9204 | **VAVQPGPAGPP**GKTPNIT |
|  | TGSDFVAVQPGPAGP | HLA-DRB1*08:02 | 0.9142 |  |
|  | TWYGIVADKDTPGGF | HLA-DRB1*04:01 | 0.9129 |  |
|  | DSASELIPIKDTLDE | HLA-DRB1*04:05 | 0.9108 |  |
|  | SELIPIKDTLDESDK | HLA-DRB1*04:05 | 0.9106 |  |
|  | YGIVADKDTPGGFAA | HLA-DRB1*04:01 | 0.908 |  |
| Gene 28  Minor Tail Protein | IQMYRINTDAQNVVG | HLA-DRB3*02:02 | 0.9517 |  |
|  | TDGVFIKVGGASLAQ | HLA-DRB1*01:01 | 0.9493 |  |
|  | DGVFIKVGGASLAQF | HLA-DRB1*01:01 | 0.9448 |  |
|  | **NNFWGALVGRQTDDD** | HLA-DRB1*01:01 | 0.9382 | **GRQTDDD**VSLADPTEQI |
|  | IRLWVRVNDDADTAG | HLA-DRB1*04:05 | 0.9356 |  |
|  | AVKFTADGTIADLYS | HLA-DRB1*04:05 | 0.9338 |  |
|  | EAPLPFILTGGQHIR | HLA-DRB1*07:01 | 0.9291 |  |
|  | SIQMYRINTDAQNVV | HLA-DRB3*02:02 | 0.9282 |  |
|  | GDVYFAEVDANKGDE | HLA-DRB1*04:01 | 0.9268 |  |
|  | LDDWVELTGTYTVPS | HLA-DRB1*01:01 | 0.9261 |  |
|  | APLPFILTGGQHIRL | HLA-DRB1*07:01 | 0.9242 |  |
|  | AVKFTADGTIADLYS | HLA-DRB1*04:01 | 0.9202 |  |
|  | LTNIVNIPTVPGTNV | HLA-DRB1*04:01 | 0.9155 |  |
|  | GAVKFTADGTIADLY | HLA-DRB1*04:05 | 0.9154 |  |
|  | GTFVRTDPADERTET | HLA-DRB1*03:01 | 0.9142 |  |
|  | **QLTNIVNIPTVPGTN** | HLA-DRB1*04:01 | 0.9129 | **TVPGTN**VGGVGG |
|  | NVNLVSDPVTTIIGD | HLA-DRB3*01:01 | 0.9113 |  |
|  | HIRLWVRVNDDADTA | HLA-DRB1*04:05 | 0.9097 |  |
|  | GVVYAYNRATQFPFN | HLA-DRB3*02:02 | 0.9071 |  |
|  | TGTFVRTDPADERTE | HLA-DRB1*03:01 | 0.9004 |  |
